# RNA m6A modification-regulated ferroptosis in cancer: mechanism and therapeutic potential

**DOI:** 10.3389/fcell.2026.1928071

**Published:** 2026-09-03

**Authors:** Qingmiao Shi, Yangni Li, Chaonan Guan

**Affiliations:** 1 Department of Infectious Diseases, The First Affiliated Hospital, College of Clinical Medicine, Henan University of Science and Technology, Luoyang, Henan, China; 2 Department of General Practice, The First Affiliated Hospital, Zhejiang University School of Medicine, Hangzhou, Zhejiang, China; 3 Translational Medicine Center, The First Affiliated Hospital of Zhengzhou University, Zhengzhou, Henan, China

**Keywords:** cancer, epitranscriptomics, ferroptosis, RNA N6-methyladenosine, therapy resistance

## Abstract

Ferroptosis, an iron-dependent form of regulated cell death, is increasingly recognized as a context-dependent therapeutic vulnerability in cancer, particularly as malignant cells adapt to oxidative, metabolic, and therapy-induced stresses. As a prevalent and reversible epitranscriptomic modification, RNA N6-methyladenosine (m6A) modification orchestrates RNA stability, translation, splicing and decay; consequently, its dysregulation contributes to cancer progression and therapeutic resistance. The intersection of m6A regulation and ferroptosis is therefore biologically important because many ferroptosis threshold genes are short-lived, stress-responsive transcripts controlled by writers, erasers, readers and RNA-binding proteins. This review synthesizes empirical evidence elucidating how m6A regulators remodel cystine import, GPX4-dependent antioxidant defense, FSP1 signaling, lipid metabolism, iron handling, autophagy and tumor-microenvironmental communication. We organize the evidence by regulatory layer rather than cancer type, covering writer-mediated deposition, reader and RNA-binding protein recognition, eraser-dependent demethylation, non-coding RNA and exosomal regulation, and downstream ferroptosis modules. We further discuss how this axis contributes to radiotherapy, chemotherapy, targeted-therapy resistance and ferroptosis-sensitizing combinations. Although m6A-ferroptosis crosstalk offers promising biomarker and therapeutic opportunities, translation requires transcript-level validation, standardized ferroptosis assays, tumor-selective delivery and clinically meaningful patient stratification. A deeper and more precise integration of epitranscriptomics with ferroptosis biology holds the potential to transform stress-adaptive RNA circuits into actionable vulnerabilities for precision cancer therapy.

## 1 Introduction

Ferroptosis has moved from a cell-death concept to a translational framework for understanding why malignant cells survive oxidative, metabolic and therapeutic stress. Unlike apoptosis or necroptosis, ferroptosis is driven by iron-dependent lipid peroxidation and by the collapse of antioxidant systems that normally restrain phospholipid damage, making it closely connected to tumor metabolism, redox adaptation and drug response (Wahida and Conrad, 2025). Cancer therapy increasingly exploits these liabilities because ferroptosis can bypass canonical apoptosis resistance and can expose vulnerabilities created by oncogenic metabolism, radiotherapy, chemotherapy and targeted therapy pressure (Zhou Q. et al., 2024). At the same time, ferroptosis is not simply a death switch, because its induction or suppression is shaped by tumor lineage, nutrient availability, lipid composition, immune context and normal-tissue tolerance (Lei et al., 2022).

RNA N6-methyladenosine (m6A) modification offers a mechanistic layer through which cancer cells can rapidly remodel these ferroptosis thresholds. The writer-reader-eraser system modulates RNA splicing, export, stability, translation and decay, thereby allowing transcript-specific control without requiring permanent genomic alteration (Deng et al., 2023). In cancer, RNA modifications also influence therapeutic resistance by stabilizing adaptive transcripts, reshaping stress-response networks and tuning the expression of drug-resistance determinants (Chen et al., 2024). This makes m6A biology particularly relevant to ferroptosis, because ferroptotic sensitivity depends on short-lived and stress-responsive transcripts encoding cystine transporters, antioxidant enzymes, lipid enzymes, iron-handling proteins and metabolic regulators.

The biological importance of the m6A-ferroptosis axis is strengthened by its links to the tumor microenvironment. RNA methylation contributes to tumor immunity and immunotherapy responsiveness by regulating immune-cell programs, cytokine pathways and checkpoint-associated phenotypes (Li et al., 2024c). Ferroptosis likewise interacts with immune surveillance, because lipid peroxidation products and ferroptotic tumor-cell states can alter antigen presentation, inflammatory signaling and immune-cell function (Bell et al., 2024). These converging observations suggest that m6A-dependent ferroptosis control may influence not only autonomous cancer-cell survival but also the response of tumors to immune and stromal pressure.

Despite rapid progress, the field remains fragmented. Many studies identify a single m6A regulator and a single ferroptosis-related target in one cancer model, but the same regulator can promote ferroptosis in one setting and suppress it in another. Broad reviews have described RNA modifications in cancer and programmed cell death, yet the specific logic that connects m6A-mediated RNA fate to ferroptosis modules across cancers still needs synthesis (Liu et al., 2022; Wu et al., 2025). A second gap is translational: ferroptosis induction is attractive, but clinical implementation requires patient selection, robust assays, tumor-selective delivery and protection of ferroptosis-sensitive normal tissues (Mao et al., 2026).

This review therefore asks how RNA m6A modification remodels ferroptosis thresholds in cancer and how this epitranscriptomic control can be translated into biomarkers, treatment-sensitization strategies and combination therapy. We first summarize m6A regulatory machinery and ferroptosis modules, then synthesize core wet-experiment-supported evidence by writers, readers, erasers, non-coding RNAs and microenvironmental networks. We finally discuss clinical stratification, therapy response, therapeutic targeting and barriers to translation, with a focus on mechanism-led patterns rather than cancer-type cataloguing.

## 2 RNA m6A modification and ferroptosis in cancer

**FIGURE 1:**
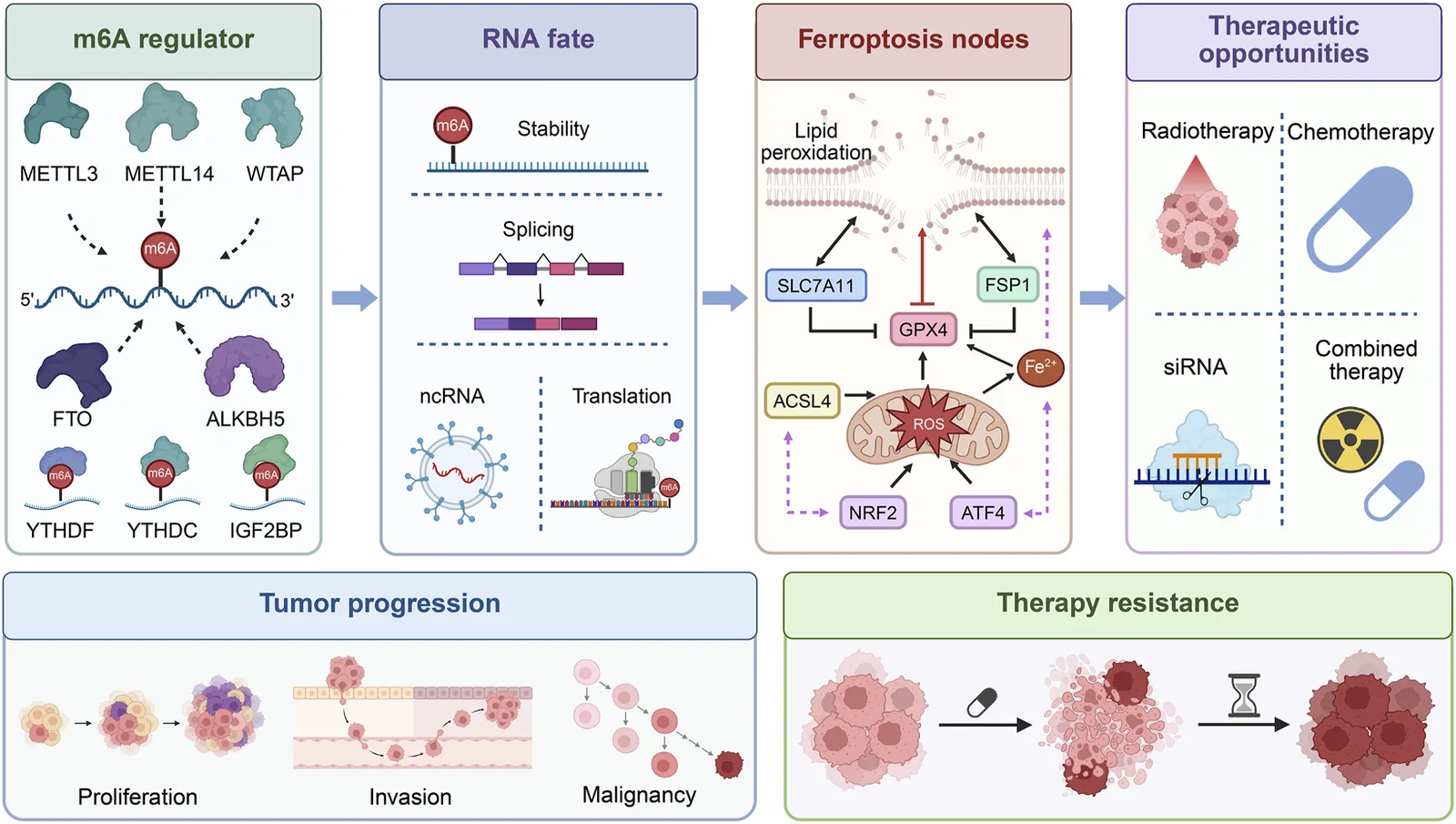
Overview of the m6A-ferroptosis axis in cancer. The figure summarizes the hierarchical regulation linking m6A machinery to ferroptosis and cancer phenotypes. Writers, erasers and readers regulate the fate of methylated RNAs through changes in RNA stability, splicing, translation and non-coding RNA function. These RNA-fate changes converge on major ferroptosis-related nodes, including SLC7A11, GPX4, FSP1, ACSL4, NRF2 and ATF4, as well as iron metabolism, reactive oxygen species (ROS) and lipid peroxidation. The resulting shift in ferroptotic susceptibility can influence tumor proliferation, invasion and malignancy, as well as therapy resistance and therapeutic response. Arrows indicate the overall direction of regulatory or functional relationships and do not necessarily represent direct molecular interactions.

RNA m6A-ferroptosis crosstalk is organized by the interaction between RNA-fate machinery and ferroptosis execution modules. Writers, erasers and readers regulate transcript stability, splicing, translation and non-coding RNA signaling, while cystine import, glutathione metabolism, lipid peroxidation, iron handling and stress-response pathways determine ferroptotic susceptibility in malignant cells (Figure 1). Together, these processes define the regulatory vocabulary needed to interpret cancer-specific m6A-ferroptosis mechanisms.

### 2.1 Overview of RNA m6A modification

m6A is a reversible RNA modification that enables transcriptome-level regulation of gene expression. Rather than functioning as a uniform mark, m6A affects RNA fate according to its location, local sequence context, RNA-binding proteins and cellular state (Roundtree et al., 2017). The canonical regulatory model divides the system into methyltransferase writers, demethylase erasers, reader proteins and RNA-binding proteins (RBPs) that translate the mark into biochemical output (Zaccara et al., 2019). This architecture is well suited to cancer stress adaptation because it can change the abundance or translation of selected transcripts faster than genomic selection.

The foundational transcriptome-mapping studies established that m6A is abundant in mammalian messenger RNAs (mRNAs) and enriched near stop codons and 3′untranslated regions, regions that are central to RNA stability and translation control (Dominissini et al., 2012; Meyer et al., 2012). The METTL3-METTL14 complex forms the core catalytic writer module, whereas associated proteins such as WTAP, VIRMA and additional adaptors help determine transcript selection and deposition patterns (Liu et al., 2014). The eraser arm was established by identification of FTO and ALKBH5 as RNA demethylases, which showed that m6A is not a static decoration but part of a reversible regulatory system (Jia et al., 2011; Zheng et al., 2013).

Readers convert methylation into functional consequence. YTH-domain proteins can recruit decay or translation machinery, IGF2BP proteins often stabilize methylated transcripts, and heterogeneous nuclear ribonucleoproteins or other RBPs can couple m6A to splicing, structure and nuclear processing. The demonstration that YTHDF2 binds m6A-marked transcripts and accelerates mRNA decay provided a model for how a single reader can impose transcript-specific turnover (Wang et al., 2014). In ferroptosis biology, this reader logic matters because many threshold-setting transcripts, including transporters and antioxidant enzymes, are sensitive to small changes in stability, splicing or translation.

### 2.2 Regulatory mechanism of ferroptosis

Ferroptosis was originally defined as an iron-dependent, nonapoptotic cell-death process triggered by lethal lipid peroxidation (Dixon et al., 2012). Its core biochemical problem is the balance between production of oxidizable phospholipids and the antioxidant systems that detoxify lipid hydroperoxides. System Xc-, the cystine/glutamate antiporter composed of solute carrier family 7 member 11 (SLC7A11) and solute carrier family 3 member 2 (SLC3A2), imports cystine to sustain glutathione synthesis; glutathione peroxidase 4 (GPX4) uses glutathione to reduce lipid peroxides; and loss of this axis exposes cells to ferroptotic collapse (Yang et al., 2014). This explains why cancer cells with high oxidative metabolism, dependency on cystine import or remodeling of polyunsaturated lipids can become ferroptosis-sensitive.

Ferroptosis is also shaped by parallel protective systems. Ferroptosis suppressor protein 1 (FSP1) suppresses ferroptosis independently of GPX4 by regenerating reduced coenzyme Q10 at the plasma membrane, giving cancer cells a second antioxidant shield (Doll et al., 2019). A related study established that FSP1 acts in parallel to GPX4, explaining why GPX4 inhibition alone may not uniformly kill all tumor cells (Bersuker et al., 2019). The GCH1-BH4 pathway adds another anti-ferroptotic layer by remodeling lipid composition and limiting lipid peroxidation (Kraft et al., 2020). Mitochondrial DHODH can also defend against ferroptosis, linking mitochondrial metabolism to cancer vulnerability (Mao et al., 2021).

The lipid substrate side is equally important. acyl-CoA synthetase long-chain family member 4 (ACSL4) promotes ferroptosis sensitivity by shaping membranes toward polyunsaturated fatty-acid-containing phospholipids, whereas oxidized arachidonic and adrenic phosphatidylethanolamines can function as execution-relevant lipid signals (Doll et al., 2017; Kagan et al., 2017). Modern ferroptosis models therefore treat the process as a metabolic network involving iron handling, lipid remodeling, antioxidant capacity, mitochondrial activity and stress signaling rather than as a single-gene phenotype (Jiang et al., 2021). This network view is crucial for interpreting m6A studies, because epitranscriptomic regulation can target any node in the network and can produce opposite phenotypes depending on which node dominates in a given tumor.

### 2.3 Linking RNA m6A modification to ferroptosis

The conceptual bridge between m6A and ferroptosis is RNA fate. m6A regulators can alter ferroptosis by changing the stability, translation, splicing or non-coding RNA function of transcripts encoding SLC7A11, SLC3A2, GPX4, FSP1, acyl-CoA synthetase long-chain family member (ACSL) enzymes, nuclear factor erythroid 2-related factor 2 (NRF2), activating transcription factor 4 (ATF4), iron-storage proteins and autophagy regulators. Studies focused on m6A and ferroptosis have proposed that this axis is particularly relevant to cancer progression and immunotherapy because it links stress-adaptive RNA control to lipid-peroxidation tolerance (Shi et al., 2024). A separate synthesis framed the m6A-ferroptosis interplay as a therapeutic avenue, but also emphasized that evidence remains heterogeneous across regulators and cancer models (Roshan et al., 2025).

This framework changes how individual studies should be interpreted. A writer such as METTL3 is not intrinsically pro-survival or pro-death; its effect depends on the methylated substrate, and the reader recruited to that substrate and the ferroptosis module that becomes limiting. Likewise, eraser inhibition may either destabilize a pro-survival transcript or preserve a pro-ferroptotic transcript, depending on whether demethylation normally protects or exposes the target RNA. Epigenetic regulation of programmed cell death therefore requires target-level validation, because the same upstream enzyme can feed into distinct death programs and signaling modules (Zhou S. et al., 2024). The practical implication is that m6A-ferroptosis research should report not only regulator expression but also methylation sites, reader dependency, transcript fate, ferroptosis rescue and therapeutic context (Figure 1).

## 3 RNA m6A modification regulates ferroptosis in cancers

**FIGURE 2:**
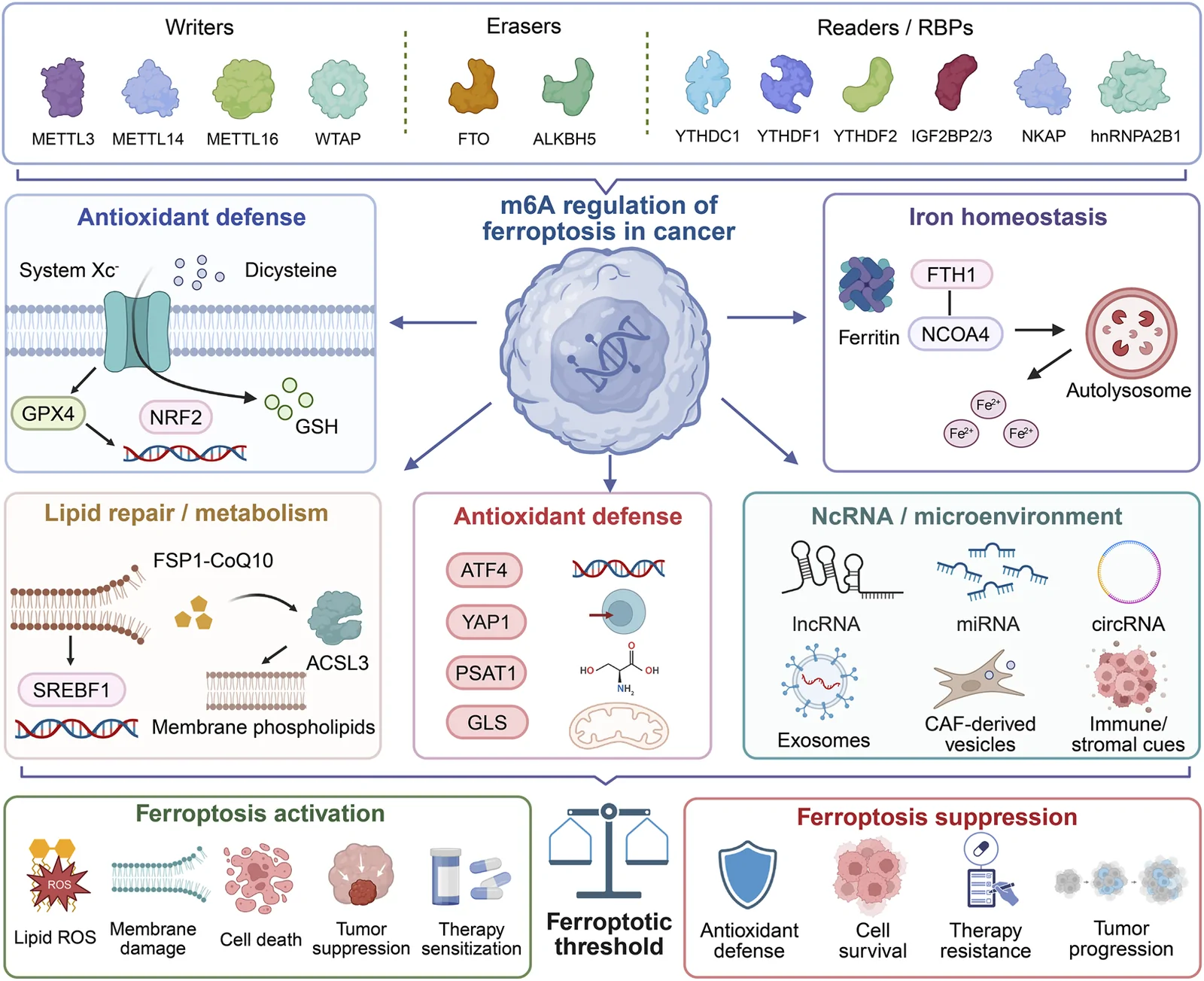
Mechanisms by which m6A regulates ferroptosis in cancer. m6A writers, erasers and readers/RNA-binding proteins regulate ferroptotic susceptibility by converging on several recurrent functional modules. These include cystine-dependent antioxidant defense through system Xc^−^ and GPX4, lipid remodeling and repair involving FSP1–CoQ10 and ACSL-family pathways, iron homeostasis and ferritinophagy involving FTH1 and NCOA4, stress-adaptive metabolic programs involving ATF4, YAP1, PSAT1 and GLS, and ncRNA- or microenvironment-mediated signaling. The net interaction among these modules determines the ferroptotic threshold of cancer cells. The lower panels illustrate the opposing biological outcomes of ferroptosis activation, characterized by lipid ROS accumulation and membrane damage, and ferroptosis suppression, which favors cell survival, therapy resistance and tumor progression. Arrows denote functional connections between regulatory layers and downstream ferroptosis modules.

RNA m6A modification regulates ferroptosis-associated tumor phenotypes through tumor-specific control of antioxidant defense, lipid metabolism, iron homeostasis, stress adaptation and non-coding RNA communication. These modules influence tumor growth, metastatic potential and treatment response by shifting the balance between lipid-peroxidation injury and ferroptosis resistance (Figure 2). Representative mechanistic studies link specific regulators, cancer types, targets and evidence systems to these recurring functional modules (Table 1).

**TABLE 1:** Core m6A regulators linking RNA modification to ferroptosis in cancer.

Types of regulators	m6A regulator	Cancer type	Target	Regulation axis	Evidence basis	Cell lines	References
Writer	WTAP	HCC	GLS; GAC	WTAP/GLS; GAC/ferroptosis resistance/suppression	Clinical samples/cohort; animal model; cell experiments; molecular/omics assays	Hep3B, LN229, U251, U87	Zhu et al. (2026)
Writer + Reader/RBP	YTHDC2; WTAP	HCC	ferroptosis-related RNA/pathway	YTHDC2; WTAP/ferroptosis-related RNA/pathway/ferroptosis modulation	Animal model; cell experiments; molecular/omics assays	HepG2, Huh7	Li et al. (2024b)
Writer	METTL14; METTL3	CRC	SLC7A11; GPx3	METTL14; METTL3/SLC7A11; GPx3/ferroptosis resistance/suppression	Clinical samples/cohort; animal model; cell experiments; molecular/omics assays	SW480	Wang et al. (2026)
Writer + Reader/RBP	METTL3; NKAP	Glioblastoma	SLC7A11; GAC	METTL3; NKAP/SLC7A11; GAC/ferroptosis modulation	Animal model; cell experiments; molecular/omics assays	U251, U87MG	(Sun et al., 2022)
Writer + Reader/RBP	METTL3; YTHDF1	LUAD	SLC7A11	METTL3; YTHDF1/SLC7A11/ferroptosis resistance/suppression	Clinical samples/cohort; animal model; cell experiments; molecular/omics assays	A549, BEAS-2B, H1299, H1975, H460, PC9	Xu et al. (2022)
Writer + Reader/RBP	YTHDF1; WTAP	Bladder cancer	NRF2; Nrf2	YTHDF1; WTAP/NRF2; Nrf2/ferroptosis modulation	Clinical samples/cohort; cell experiments	—	Wang et al. (2023b)
Writer	METTL16	Cholangiocarcinoma	ATF4	METTL16/ATF4/ferroptosis resistance/suppression	Clinical samples/cohort; animal model; cell experiments; molecular/omics assays	RBE	Zhao et al. (2025)
Writer + Reader/RBP	METTL3; YTHDF2	Hepatoblastoma	ATF4; YAP1; PSAT1; LATS2	METTL3; YTHDF2/ATF4; YAP1; PSAT1; LATS2/ferroptosis induction/sensitization	Molecular/omics assays; cell experiments	HepG2	Zhu et al. (2024)
Writer	METTL14	NSCLC	SLC3A2	METTL14/SLC3A2/ferroptosis modulation	Clinical samples/cohort; animal model; cell experiments; molecular/omics assays	A549, BEAS-2B, H1299, H460	Chen et al. (2025)
Writer + Reader/RBP	METTL14; YTHDF1	NSCLC	GPX4; PKM2	METTL14; YTHDF1/GPX4; PKM2/ferroptosis modulation	Clinical samples/cohort; animal model; cell experiments	—	Liu et al. (2025b)
Writer	METTL7B	Bladder cancer	ACSL3	METTL7B/ACSL3/ferroptosis modulation	Animal model; cell experiments; molecular/omics assays	5637, T24, UM-UC-3	He et al. (2025)
Reader/RBP	YTHDC2	LUAD	SLC7A11	YTHDC2/SLC7A11/ferroptosis resistance/suppression	Animal model; cell experiments	A549, BEAS-2B, H1299, H1650, H1975, HCC827	Ma et al. (2021a)
Reader/RBP	YTHDC2	LUAD	SLC7A11; SLC3A2	YTHDC2/SLC7A11; SLC3A2/ferroptosis induction/sensitization	Clinical samples/cohort; animal model; cell experiments; molecular/omics assays	—	Ma et al. (2021b)
Reader/RBP	YTHDC1	Pan-cancer/cancer model	GPX4; FSP1	YTHDC1/GPX4; FSP1/ferroptosis resistance/suppression	Cell experiments	—	Yuan et al. (2023)
Reader/RBP	IGF2BP3	Glioma	GPX4	IGF2BP3/GPX4/ferroptosis induction/sensitization	Animal model; cell experiments; molecular/omics assays	U251, U87	Deng et al. (2024)
Reader/RBP	IGF2BP2	CRC	NRF2; Nrf2	IGF2BP2/NRF2; Nrf2/ferroptosis resistance/suppression	Animal model; cell experiments; molecular/omics assays	HCT116, HT29, LoVo, SW480, SW620	Zhu et al. (2025a)
Reader/RBP	IGF2BP3	Ovarian cancer	CACNA1A	IGF2BP3/CACNA1A/ferroptosis modulation	Cell experiments; molecular/omics assays	A2780, SKOV3	Gong et al. (2025)
Reader/RBP	HNRNPA2B1	Breast cancer	ACSL4	HNRNPA2B1/ACSL4/ferroptosis induction/sensitization	Cell experiments; molecular/omics assays	—	Yuan et al. (2026)
Reader/RBP	YTHDF1	Lung cancer	ferroptosis-related RNA/pathway	YTHDF1/ferroptosis-related RNA/pathway/ferroptosis induction/sensitization	Cell experiments	A549, H1299	Diao et al. (2023)
Reader/RBP	YTHDF1	Pan-cancer/cancer model	SLC7A11; GPX4; FTH1	YTHDF1/SLC7A11; GPX4; FTH1/ferroptosis induction/sensitization	Cell experiments	BEAS-2B	Wang et al. (2024)
Eraser + Reader/RBP	YTHDF2; FTO	CRC	SLC7A11; GPX4	YTHDF2; FTO/SLC7A11; GPX4/ferroptosis induction/sensitization	Clinical samples/cohort; cell experiments	HCT116, LoVo	Qiao et al. (2024)
Eraser + Reader/RBP	YTHDF2; FTO	CRC	GPX4	YTHDF2; FTO/GPX4/ferroptosis induction/sensitization	Animal model; cell experiments; molecular/omics assays	SW480	Zhang et al. (2023)
Eraser	FTO	Glioblastoma	SLC7A11	FTO/SLC7A11/ferroptosis resistance/suppression	Clinical samples/cohort; cell experiments; Molecular/omics assays	LN229, U87	Tian et al. (2025)
Eraser	ALKBH5	CRC	SLC7A11	ALKBH5/SLC7A11/ferroptosis resistance/suppression	Molecular/omics assays	—	Qu et al. (2025)
Eraser	FTO	Thyroid carcinoma	SLC7A11	FTO/SLC7A11/ferroptosis modulation	Clinical samples/cohort; animal model; molecular/omics assays; cell experiments	KTC-1, TPC-1	Ji et al. (2022)
Eraser	FTO	OSCC	GPX4; ACSL3	FTO/GPX4; ACSL3/ferroptosis resistance/suppression	Animal model; cell experiments	SCC-25	Wang et al. (2023c)
Reader/RBP	YTHDF2	Gastric cancer	ACSL4; CBS	YTHDF2/ACSL4; CBS/ferroptosis induction/sensitization	Clinical samples/cohort; animal model; cell experiments	AGS, MKN45	Yang et al. (2022)
Reader/RBP	SND1	Glioma	NRF2; Nrf2	SND1/NRF2; Nrf2/ferroptosis induction/sensitization	Animal model; cell experiments; molecular/omics assays	A172, LN229, T98G, U251, U373, U87MG	Zheng et al. (2023)
Writer + Reader/RBP	IGF2BP2; METTL3	CRC	FOXM1	IGF2BP2; METTL3/FOXM1/ferroptosis resistance/suppression	Clinical samples/cohort; animal model; cell experiments; Molecular/omics assays	—	Bian et al. (2024)
Reader/RBP	IGF2BP2	Glioma	GPX4	IGF2BP2/GPX4/ferroptosis induction/sensitization	Clinical samples/cohort; animal model; cell experiments	—	Yang et al. (2023)
Eraser	ALKBH5	LUAD	GPX4	ALKBH5/GPX4/ferroptosis resistance/suppression	Clinical samples/cohort; animal model; cell experiments; molecular/omics assays	A549, H1299, H1975, H358, PC9	Ge et al. (2025)
Writer + Reader/RBP	METTL14; YTHDC2	Ovarian cancer	SLC7A11	METTL14; YTHDC2/SLC7A11/ferroptosis resistance/suppression	Clinical samples/cohort; animal model; cell experiments	A2780, OVCAR3, SKOV3	Yang et al. (2025a)
Reader/RBP	YTHDF2	LUAD	SLC2A3	YTHDF2/SLC2A3/ferroptosis induction/sensitization	Animal model; cell experiments	—	Xu et al. (2025)
Writer	METTL3	CRC	ACSL3	METTL3/ACSL3/ferroptosis resistance/suppression	Animal model; molecular/omics assays; cell experiments	LoVo	(Ren et al., 2024)
Writer	METTL3	Glioblastoma	NCOA4	METTL3/NCOA4/ferroptosis induction/sensitization	Clinical samples/cohort; animal model; cell experiments; Molecular/omics assays	A172, T98G, U251, U87MG	Zhou et al. (2026)
Other/Unspecified m6A-related	m6A-related regulator	Breast cancer	SLC7A11; FGFR4	m6A-related regulator/SLC7A11; FGFR4/ferroptosis modulation	Animal model; cell experiments	MCF-7, MDA-MB-231, MDA-MB-453, SKBR3, T47D	Zou et al. (2022)
Other/Unspecified m6A-related	not explicitly specified	NSCLC	GPX4	not explicitly specified/GPX4/ferroptosis resistance/suppression	Clinical samples/cohort; animal model; cell experiments	A549, BEAS-2B, H1299, H1975	Liu et al. (2025a)

### 3.1 m6A writers and ferroptosis

Writer-mediated evidence is the densest part of the field, and it shows that m6A deposition remodels ferroptosis through substrate-specific control rather than through a uniform writer phenotype. In hepatocellular carcinoma (HCC), WTAP-mediated splicing bias of glutaminase favored the GAC isoform and suppressed ferroptosis, connecting m6A machinery to glutamine metabolism and redox tolerance (Zhu et al., 2026). A second HCC study linked WTAP-associated m6A regulation to autophagy-dependent ferroptosis control, suggesting that writer complexes can connect RNA methylation to organelle degradation as well as to canonical antioxidant genes (Li et al., 2024b). These findings place metabolic flux and autophagy beside SLC7A11 and GPX4 as writer-regulated ferroptosis determinants.

METTL3 and METTL14 frequently converge on cystine import and antioxidant defense. In colorectal cancer (CRC), METTL3/14-mediated m6A silencing of GPx3 promoted lipophagy dysfunction and ferroptosis resistance, showing that writer activity can affect both lipid turnover and peroxide detoxification (Wang et al., 2026). In glioblastoma, NKAP protected cells from ferroptosis by promoting SLC7A11 mRNA splicing in an m6A-dependent manner, illustrating that writer-associated RBPs can determine ferroptosis by processing rather than simply stabilizing transcripts (Sun et al., 2022). METTL3 also stabilized SLC7A11 in lung adenocarcinoma and supported tumor growth while inhibiting ferroptosis, reinforcing cystine transport as one of the recurrent m6A-sensitive nodes (Xu et al., 2022).

Antioxidant transcriptional programs add another layer of writer control. In bladder cancer, WTAP targeted NRF2 and accelerated malignancy through m6A-dependent ferroptosis regulation, linking writer activity to a major oxidative-stress transcription factor (Wang K. et al., 2023). In cholangiocarcinoma, METTL16 promoted ATF4 through m6A modification and suppressed ferroptosis, placing integrated stress-response signaling within the same regulatory logic (Zhao et al., 2025). In hepatoblastoma, m6A modification of LATS2 promoted progression by inhibiting ferroptosis through the YAP1/ATF4/PSAT1 axis, indicating that writer-mediated ferroptosis control can be embedded in developmental and amino-acid metabolism pathways (Zhu et al., 2024).

The writer layer also includes noncanonical methyltransferase-like proteins and context-specific targets. METTL14 enhanced cell ferroptosis through the SLC3A2 axis in non-small cell lung cancer (NSCLC) when JMJD6 K375 acetylation restrained tumor progression, suggesting that upstream post-translational regulation can redirect m6A-ferroptosis output (Chen et al., 2025). SETD5 facilitated stemness and repressed ferroptosis by stabilizing PKM2 through an m6A-related mechanism, connecting glycolytic reprogramming to GPX4-linked resistance in NSCLC (Liu X. et al., 2025). In bladder cancer, METTL7B participated in tumor progression through ACSL3 m6A modification, extending writer-linked ferroptosis control to lipid-acylation enzymes (He et al., 2025).

The context-dependent effects of METTL3 illustrate why writer activity cannot be interpreted independently of its RNA targets. METTL3 can reinforce ferroptosis resistance when its downstream regulation preserves cystine import or antioxidant and lipid-survival programs, as observed for SLC7A11-and ACSL3-associated pathways. By contrast, METTL3 can also favor ferroptotic vulnerability when its activity is coupled to NCOA4-dependent iron mobilization and ferritinophagy. These apparently opposing outcomes therefore reflect the ferroptosis module engaged by the dominant target transcript rather than an intrinsically pro- or anti-ferroptotic function of METTL3. Reader availability, upstream signaling and the metabolic state of the tumor may further determine how a given m6A event is translated into RNA fate and ferroptosis sensitivity.

### 3.2 m6A readers and ferroptosis

Readers and RBPs are the output layer that turns m6A deposition into ferroptosis phenotypes. YTHDC2 suppressed lung adenocarcinoma (LUAD) tumorigenesis by limiting SLC7A11-dependent antioxidant function, identifying an endogenous reader-mediated route to ferroptosis induction (Ma et al., 2021a). A related LUAD study showed that targeting the SLC3A2 subunit of system Xc-was essential for YTHDC2 to function as a ferroptosis inducer, narrowing the reader effect to a defined cystine-import module (Ma et al., 2021b). In contrast, YTHDC1 acted as a tumor suppressor by modulating FSP1-dependent ferroptosis suppression in lung cancer, indicating that nuclear reader pathways can influence a GPX4-independent antioxidant system (Yuan et al., 2023).

IGF2BP-family proteins often stabilize pro-survival ferroptosis transcripts. Depletion of IGF2BP3 induced ferroptosis in glioma by modulating GPX4 expression, supporting the idea that reader-dependent stabilization can preserve antioxidant defenses (Deng et al., 2024). In colorectal cancer, histone lactylation upregulated IGF2BP2 and enhanced ferroptosis resistance through NRF2, linking metabolic epigenetic marks, m6A reader expression and oxidative-stress buffering (Zhu J. F. et al., 2025). In ovarian cancer, m6A-mediated stabilization of CACNA1A by IGF2BP3 drove progression by inhibiting ferroptosis, expanding reader control beyond classical ferroptosis genes into calcium-channel-associated regulatory circuits (Gong et al., 2025).

Other RNA-binding proteins broaden the reader concept beyond YTH and IGF2BP proteins. circSCYL2 antagonized hnRNPA2B1-mediated m6A-chromatin crosstalk and promoted ferroptosis while inhibiting breast-cancer metastasis, suggesting that m6A-linked RBPs can integrate RNA processing with chromatin state and metastatic behavior (Yuan et al., 2026). YTHDF1 promoted lung carcinoma progression through regulation of ferritin-mediated ferroptosis, showing that reader-driven translation can affect iron-storage biology (Diao et al., 2023). Benzo(a)pyrene also promoted malignant progression of transformed bronchial epithelial cells through YTH-family regulation of SLC7A11, GPX4 and FTH1, connecting environmental carcinogen exposure to reader-mediated ferroptosis resistance (Wang et al., 2024).

Reader-dependent regulation shows a similar pattern of convergence. Although different readers act through distinct RNA-fate mechanisms, their downstream effects repeatedly involve cystine transport, GPX4/FSP1-dependent antioxidant defense, lipid metabolism and iron handling. Which reader is recruited to a methylated transcript can therefore determine not only its stability or translation but also which ferroptosis-defense module becomes functionally dominant.

### 3.3 m6A erasers and ferroptosis

Eraser studies are fewer but especially important because they expose the reversibility of ferroptosis control. Targeting FTO induced ferroptotic cell death in colorectal cancer by decreasing SLC7A11 and GPX4 expression, suggesting that FTO activity can maintain antioxidant capacity in certain CRC contexts (Qiao et al., 2024). Another CRC study showed that AKT targeting induced ferroptosis through an FTO/YTHDF2-dependent GPX4 m6A mechanism, placing demethylation within a kinase-regulated therapeutic axis (Zhang et al., 2023). In glioblastoma, IDO1 inhibited ferroptosis by regulating FTO-mediated m6A methylation and SLC7A11 mRNA stability, linking immune-metabolic enzyme activity to epitranscriptomic cystine-import control (Tian et al., 2025).

ALKBH5 and FTO are not functionally equivalent. PRMT5 K240 lactylation conferred ferroptosis resistance through the ALKBH5/SLC7A11 axis in colorectal cancer, suggesting that post-translational modification of upstream chromatin or methylation regulators can reshape demethylase-dependent ferroptosis output (Qu et al., 2025). In thyroid carcinoma, FTO prevented tumor progression through SLC7A11 m6A methylation in a ferroptosis-dependent manner, contrasting with studies where FTO supports ferroptosis resistance and underscoring the need for target-level validation (Ji et al., 2022). In oral squamous cell carcinoma (OSCC), FTO sensitized cells to ferroptosis by suppressing ACSL3 and GPX4, again showing that eraser activity can either protect or expose tumors depending on the dominant target transcripts (Wang Z. et al., 2023).

These apparently divergent effects suggest that the ferroptotic consequence of FTO activity is determined less by the demethylase itself than by the RNA substrates and ferroptosis modules that predominate in a given tumor context. In colorectal cancer, FTO activity is associated with maintenance of the SLC7A11/GPX4 antioxidant axis, whereas in thyroid carcinoma and OSCC, FTO-dependent regulation is linked to increased ferroptotic susceptibility through SLC7A11-or ACSL3/GPX4-related mechanisms. Such context-dependent effects may reflect differences in the spectrum and location of m6A-modified target transcripts, the availability of reader proteins that determine subsequent RNA fate, and the relative dependence of individual tumors on cystine import, GPX4-mediated antioxidant defense, or lipid-metabolic pathways. Upstream signaling and the basal metabolic and redox state may further influence which FTO-regulated transcript becomes functionally dominant. However, because these studies were conducted in different tumor models and did not directly compare m6A sites, reader recruitment, or cellular conditions across cancer types, these factors currently provide plausible mechanistic explanations rather than established causes of the divergent FTO phenotypes.

### 3.4 Non-coding RNA-associated m6A-ferroptosis networks

Non-coding RNAs participate in m6A-ferroptosis regulation through mechanistically distinct configurations. In some cases, the ncRNA itself is reported to be regulated by m6A modification. In colorectal cancer, m6A modification of ABHD11-AS1 promoted tumor progression and ferroptosis resistance through a TRIM21/IGF2BP2/FOXM1 regulatory circuit (Bian et al., 2024). Similarly, the m6A-regulated lncRNA AC040169.1 suppressed ferroptosis through SLC7A11 in ovarian cancer (Yang H. et al., 2025). These studies more directly connect the methylation state of an ncRNA with downstream ferroptosis-related signaling.

In other ncRNA-centered networks, however, the ncRNA participates in an m6A-dependent regulatory circuit without necessarily being the principal methylated substrate. Hypoxia-inducible lncRNA CBSLR modulated ferroptosis in gastric cancer through YTHDF2-dependent regulation of CBS and ACSL4, linking hypoxic signaling to sulfur metabolism and lipid peroxidation (Yang et al., 2022). In glioma, SNAI3-AS1 promoted ferroptosis by interfering with SND1-mediated m6A recognition of NRF2 mRNA rather than simply acting as an m6A-modified ferroptosis effector itself (Zheng et al., 2023). TMEM44-AS1 was likewise linked to ferroptosis through the IGF2BP2-GPX4 axis in esophageal squamous cell carcinoma, illustrating how an ncRNA can interface with an m6A reader-dependent antioxidant pathway (Yang et al., 2023). ALKBH5-dependent regulation of RBMS3-AS3 was associated with GPX4-mediated ferroptosis resistance in lung adenocarcinoma, although the extent to which site-specific methylation of the lncRNA itself is required for this phenotype remains less clearly established (Ge et al., 2025).

Collectively, these studies reveal considerable mechanistic diversity in ncRNA-associated m6A–ferroptosis regulation. ncRNAs may either be directly influenced by m6A-dependent RNA processing or participate in reader- and target-centered regulatory circuits that ultimately converge on key ferroptosis determinants such as SLC7A11, GPX4, ACSL4 and NRF2. This diversity highlights ncRNAs as important intermediates linking epitranscriptomic regulation with antioxidant defense, lipid metabolism and cellular stress adaptation in cancer.

### 3.5 Tumor microenvironment and m6A-ferroptosis networks

The microenvironment adds another dimension. Neutrophil extracellular traps promoted lung adenocarcinoma growth by increasing the stability of m6A-mediated SLC2A3 mRNA, linking inflammatory extracellular structures to glucose uptake and ferroptosis-related survival (Xu et al., 2025). Cancer-associated fibroblast-derived exosomal METTL3 promoted colorectal cancer proliferation and metastasis while suppressing ferroptosis through ACSL3, suggesting that stromal m6A signals can be transferred into tumor-cell lipid metabolism (Ren et al., 2024). In glioblastoma, the GDNF-AS1/LHX2/METTL3/NCOA4 axis inhibited progression by inducing ferroptosis, showing that ncRNA-linked m6A regulation can also favor ferritinophagy and tumor suppression (Zhou et al., 2026). This evidence reframes m6A-ferroptosis regulation as an ecosystem property. Tumor cells, stromal cells, immune cells and extracellular vesicles can all influence RNA methylation states and ferroptosis thresholds. The challenge is to identify which signals are causal *in vivo* rather than merely correlated with aggressive disease states.

An emerging question is how m6A-regulated ferroptosis interfaces with antitumor immunity. In lung adenocarcinoma, NET-associated stabilization of m6A-modified SLC2A3 was linked not only to ferroptosis resistance but also to CD8^+^ T-cell inhibition, suggesting that tumor-cell ferroptosis programs can influence the immune microenvironment (Xu et al., 2025). More broadly, m6A-dependent RNA regulation and ferroptotic stress may intersect with the functional state of tumor-infiltrating lymphocytes, myeloid-derived suppressor cells and tumor-associated macrophages through changes in redox balance, lipid metabolism and intercellular signaling. Whether these effects arise predominantly from tumor-cell-derived signals or from cell-intrinsic m6A–ferroptosis programs within immune populations remains insufficiently defined. Resolving this crosstalk may help clarify how the m6A–ferroptosis axis contributes to immunotherapy sensitivity or resistance.

### 3.6 Convergent ferroptosis modules

Across writers, readers, erasers and ncRNAs, the same downstream modules recur. SLC7A11 and SLC3A2 determine cystine import, GPX4 and FSP1 determine peroxide detoxification, ACSL3 and ACSL4 tune lipid substrate availability, and NRF2 or ATF4 provide stress-responsive transcriptional buffering. In HER2-positive breast cancer, m6A-regulated FGFR4 attenuated ferroptotic cell death and contributed to a recalcitrant phenotype, showing that receptor-tyrosine-kinase signaling can intersect with m6A-dependent ferroptosis control (Zou et al., 2022). In NSCLC, loss of NLN suppressed tumor progression by inducing ferroptosis through down-regulation of m6A methylation of GPX4, identifying a protease-linked route into the peroxide-detoxification module (Liu L. et al., 2025).

The recurrent involvement of these downstream modules also provides a mechanistic basis for the context-dependent effects of individual m6A regulators. The same regulator may shift ferroptosis in opposite directions when different target transcripts become dominant, because cystine import, GPX4/FSP1-dependent antioxidant defense, lipid remodeling, iron handling and autophagy impose distinct rate-limiting constraints across tumor types and treatment states. The functional consequence of an m6A event therefore emerges from the combination of target selection, reader-dependent RNA fate and the ferroptosis pathway that is most limiting in a given cellular context.

## 4 Biomarker and therapeutic potential

**FIGURE 3:**
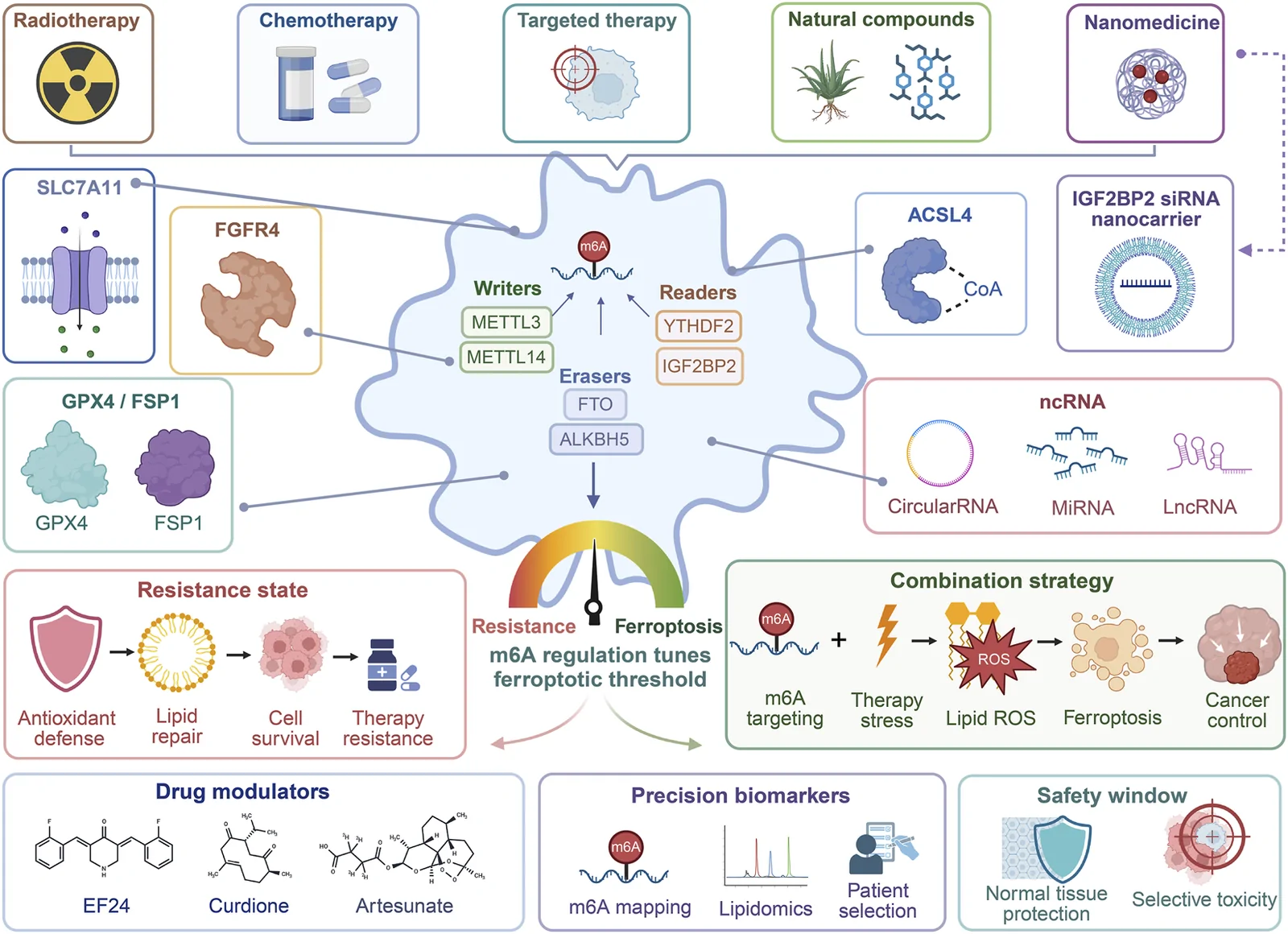
Therapeutic targeting of the m6A-ferroptosis axis. The figure illustrates how m6A-dependent ferroptosis regulation intersects with anticancer therapy and therapeutic resistance. Radiotherapy, chemotherapy, targeted therapy, natural compounds and nanomedicine can interact with m6A writers, erasers and readers to alter ferroptosis-related pathways, including SLC7A11-mediated cystine transport, GPX4/FSP1 antioxidant defense, ACSL4-dependent lipid remodeling and ncRNA-associated regulation. Shifting these pathways from an antioxidant resistance state toward lipid ROS accumulation and ferroptosis may enhance treatment response. Representative therapeutic approaches include pharmacological modulators such as EF24, curdione and artesunate, RNA-based or nanocarrier-mediated intervention, and combination strategies that couple m6A modulation with ferroptosis induction. Precision biomarkers, including m6A profiling and lipid-related molecular features, may support patient selection, whereas tumor-selective delivery and protection of normal tissues are important for maintaining an acceptable therapeutic window. Arrows indicate proposed therapeutic or regulatory relationships, and the central balance represents the shift between ferroptosis resistance and ferroptotic susceptibility.

m6A-ferroptosis circuits have translational relevance when regulator-target pairs stratify tumors, explain treatment failure or reveal druggable ferroptotic vulnerabilities. Clinical and preclinical evidence connects this axis with radiotherapy response, chemotherapy resistance, targeted-therapy adaptation, natural compound sensitivity, RNA-based delivery and biomarker-defined patient selection. These applications position the m6A-ferroptosis axis as a candidate framework for combination therapy and precision intervention (Figure 3; Table 2).

**TABLE 2:** Therapeutic contexts linked to the m6A-ferroptosis axis.

Therapeutic context	Cancer type	m6A regulator	Target	Regulation axis	Evidence basis	Cell lines	References
Radiotherapy sensitization	HCC	IGF2BP2; METTL3; YTHDF2	SLC7A11	IGF2BP2; METTL3; YTHDF2/SLC7A11/ferroptosis induction/sensitization	Clinical samples/cohort; animal model; cell experiments	Hep3B, HepG2, Huh7	Zhang et al. (2025)
Radiotherapy resistance	NPC	METTL3	SLC7A11	METTL3/SLC7A11/ferroptosis resistance/suppression	Molecular/omics assays; cell experiments	HONE1, SUNE1	Dai et al. (2025)
Cisplatin resistance	Lung cancer	METTL3	FSP1	METTL3/FSP1/ferroptosis resistance/suppression	Clinical samples/cohort; animal model; cell experiments; molecular/omics assays	A549	Song et al. (2021)
Cisplatin resistance	Esophageal cancer	METTL14	FSP1	METTL14/FSP1/ferroptosis resistance/suppression	Animal model; cell experiments; molecular/omics assays	—	Han et al. (2025)
Anlotinib resistance	Osteosarcoma	METTL3; YTHDF2; YTHDF1	GPX4; PRKDC	METTL3; YTHDF2; YTHDF1/GPX4; PRKDC/ferroptosis resistance/suppression	Animal model; cell experiments	—	Zhang et al. (2026)
HAIC resistance	HCC	IGF2BP3	FSP1; PARK7	IGF2BP3/FSP1; PARK7/ferroptosis resistance/suppression	Animal model; cell experiments	Hep3B, Huh7, SNU-398, SNU-449	Zhu et al. (2025b)
Radiotherapy resistance	Thyroid carcinoma	YTHDF2	SREBF1	YTHDF2/SREBF1/ferroptosis resistance/suppression	Animal model; cell experiments; molecular/omics assays	—	Dai et al. (2026)
Small-molecule METTL3 targeting (EF24)	Glioma	METTL3; YTHDF1	GPX4; NRF2; Nrf2	METTL3; YTHDF1/GPX4; NRF2; Nrf2/ferroptosis induction/sensitization	Clinical samples/cohort; animal model; cell experiments; molecular/omics assays	A172, HeLa, LN229	Yang et al. (2025b)
Natural compound-induced ferroptosis (Curdione)	CRC	METTL14; YTHDF2	SLC7A11; SLC3A2; GPX4	METTL14; YTHDF2/SLC7A11; SLC3A2; GPX4/ferroptosis induction/sensitization	Clinical samples/cohort; animal model; cell experiments; molecular/omics assays	CT26, SW480	Wang et al. (2023a)
Artesunate-induced ferroptosis	HCC	YTHDC2; WTAP	ferroptosis-related RNA/pathway	YTHDC2; WTAP/ferroptosis-related RNA/pathway/ferroptosis modulation	Animal model; cell experiments; molecular/omics assays	—	Li et al. (2026b)
Photothermal nanocarrier and siRNA delivery	HNSCC	IGF2BP2	SLC7A11; GPX4; NRF2	IGF2BP2/SLC7A11; GPX4; NRF2/ferroptosis modulation	Animal model; cell experiments; molecular/omics assays	CAL27, FaDu	Li et al. (2026a)
Irradiation-induced ferroptosis/radiosensitization	Oral cancer	m6A-related regulator	ACSL4	m6A-related regulator/ACSL4/ferroptosis induction/sensitization	Animal model; cell experiments; molecular/omics assays	—	Chen et al. (2026)
Sorafenib-induced ferroptosis	Cervical cancer	METTL14	FTH1	METTL14/FTH1/ferroptosis induction/sensitization	Clinical samples/cohort; animal model; cell experiments; molecular/omics assays	CaSki, HeLa, SiHa	Li et al. (2024a)

### 4.1 Development of biomarkers

The current evidence suggests that m6A-ferroptosis regulators have promising stratification potential, although their readiness for routine clinical biomarker application remains limited. Several core studies combine clinical samples with cell and animal experiments, suggesting that m6A-ferroptosis regulators can be linked to tumor aggressiveness, survival-associated expression patterns or therapy-responsive phenotypes. In glioblastoma, intracellular C5aR1 inhibited ferroptosis through METTL3-dependent m6A methylation of GPX4, providing a clinically anchored example in which an immune-complement receptor converges on a canonical antioxidant enzyme (Meng et al., 2024). In lung adenocarcinoma, downregulation of m6A-modified LINC00641 promoted epithelial-mesenchymal transition (EMT) but also created a ferroptotic vulnerability, showing that stratification may need to capture both progression state and therapy sensitivity (Xi et al., 2023). In bladder cancer, m6A-modified circCD2AP suppressed ferroptosis through a FOXC1-PARK7 circuit, linking clinical tumor behavior to a reader-associated antioxidant pathway (Wang et al., 2025). In endometrial cancer, lncRNA NORAD regulated ferroptosis by modifying GPX4 through FTO-mediated m6A control, extending the biomarker concept to eraser-lncRNA-target circuits (Ke et al., 2025).

A practical stratification model would focus on regulator-target pairs rather than isolated regulator expression. Tumors with high SLC7A11 or GPX4 dependency may respond differently to m6A modulation than tumors dominated by FSP1, ACSL3, ACSL4 or NRF2 buffering. Clinical translation will therefore require paired measurement of m6A regulators, methylated target transcripts, ferroptosis module activity and treatment context. Such paired assays are more demanding than single-gene markers, but they better reflect the biology of an RNA-fate-controlled death threshold.

Three validation steps are particularly important. First, candidate biomarkers should be tested in independent cohorts with matched treatment information rather than only in discovery samples. Second, transcript-level methylation or reader-binding readouts should be connected to functional ferroptosis markers, because regulator expression alone cannot identify the active downstream module. Third, stratification models should be designed around clinical decisions, such as whether to combine radiotherapy with ferroptosis induction, whether to add cystine-import inhibition, or whether to target reader-dependent GPX4 or FSP1 protection.

### 4.2 Application in therapy resistance

Therapy-response studies show that m6A-ferroptosis regulation becomes particularly relevant under treatment pressure. In hepatocellular carcinoma, METTL3 inhibition promoted radiosensitivity by regulating SLC7A11 expression, positioning cystine-import control as a radiosensitization mechanism (Zhang et al., 2025). In nasopharyngeal carcinoma, METTL3-mediated m6A modification of SLC7A11 enhanced radioresistance by suppressing ferroptosis, reinforcing SLC7A11 as a recurring radiation-resistance node (Dai et al., 2025).

Resistance is not limited to radiation. In lung cancer, exosomal miR-4443 promoted cisplatin resistance by regulating FSP1 through an m6A-dependent mechanism, connecting extracellular RNA transfer to GPX4-independent ferroptosis defense (Song et al., 2021). In esophageal cancer, exosomal miR-130a-3p conferred cisplatin resistance by suppressing METTL14-dependent ferroptosis regulation through FSP1, highlighting convergent use of the FSP1 shield under platinum pressure (Han et al., 2025). In osteosarcoma, METTL3-mediated m6A modification contributed to anlotinib resistance through a circFAM120B/PRKDC/GPX4 pathway, extending the axis to targeted-therapy resistance (Zhang et al., 2026).

Metabolic and regional treatment contexts further broaden the translational landscape. PARK7-driven lactylation of IGF2BP3 mediated ferroptosis and hepatic arterial infusion chemotherapy (HAIC) resistance in HCC, linking lactate-associated protein modification to reader-dependent treatment failure (Zhu Z. et al., 2025). In thyroid carcinoma, YTHDF2 stabilized SREBF1 and promoted lipid metabolic reprogramming associated with ferroptosis-related radioresistance, indicating that reader control of lipid synthesis can alter radiation response (Dai et al., 2026). These examples show that therapy-induced ferroptosis is constrained by both antioxidant defenses and metabolic rewiring.

### 4.3 Targeting strategies and challenges

Therapeutic targeting can proceed at several levels: m6A enzymes, readers/RBPs, ferroptosis execution modules, delivery systems or combination regimens. EF24 targeted METTL3 to reprogram m6A methylation and induce ferroptosis in glioma, providing an example of a small molecule that connects epitranscriptomic modulation to ferroptotic killing (Yang Y. et al., 2025). Curdione induced ferroptosis in colorectal cancer through METTL14 and YTHDF2-mediated m6A regulation, suggesting that natural compounds may exploit the same axis when mechanism and rescue evidence are available (Wang F. et al., 2023). Artesunate required WTAP/YTHDC2-linked m6A-dependent autophagy activation for anti-tumor ferroptosis in HCC, supporting combination concepts that connect autophagy, m6A and ferroptosis induction (Li Y. et al., 2026).

Delivery technologies may be essential because many m6A regulators are broadly expressed. An exosome-biomimetic photothermal nanocarrier delivered IGF2BP2 siRNA and enhanced ferroptosis in head and neck squamous cell carcinoma, illustrating a strategy for combining targeted RNA interference with ferroptosis-promoting therapy (Li M. et al., 2026). In oral cancer, deletion of GPX8 enhanced irradiation-induced ferroptosis through m6A hypomethylation-mediated upregulation of ACSL4, suggesting that manipulating upstream redox enzymes can sensitize tumors to radiotherapy by increasing lipid-peroxidation susceptibility (Chen et al., 2026). In cervical cancer, METTL14 decreased FTH1 mRNA stability and promoted sorafenib-induced ferroptosis, linking iron-storage regulation to drug sensitization (Li L. et al., 2024).

The translational barriers are substantial. First, m6A enzymes and readers regulate thousands of transcripts, so systemic inhibition may create broad RNA-fate toxicity. Second, the therapeutic window of ferroptosis induction is constrained by the susceptibility of normal tissues to lipid-peroxidation injury. Renal, hepatic and neural tissues can be particularly vulnerable under conditions of oxidative or metabolic stress, raising the possibility that systemic manipulation of m6A-regulated ferroptosis pathways may also alter ferroptosis thresholds outside the tumor. Although direct evidence for tissue-specific m6A–ferroptosis regulation in these normal compartments remains limited, this potential effect should be considered when targeting broadly expressed m6A regulators. Tumor-selective delivery, local administration and dose-sparing combination strategies may therefore be preferable to systemic modulation when developing m6A-based ferroptosis therapies. Tumor-oriented delivery platforms that preferentially increase intratumoral exposure may further help restrict the effects of m6A-directed interventions. In parallel, patient selection should move beyond the expression of individual m6A regulators and instead consider whether a defined regulator–target circuit and its corresponding ferroptosis dependency are active in a given tumor and treatment context. Third, variation in ferroptosis assays, rescue conditions and experimental models continues to limit cross-study comparison. Ferroptosis is more convincingly established when lipid peroxidation is accompanied by ferroptosis-specific rescue or evidence of iron dependence, rather than being inferred solely from changes in individual markers such as GPX4 or SLC7A11 (Mishima et al., 2025). For m6A-related mechanisms, stronger mechanistic support is provided when changes in an m6A regulator can be connected to modification of a defined target RNA, the resulting alteration in RNA fate, and the downstream ferroptosis phenotype. Such integration of epitranscriptomic and functional evidence would improve the interpretability and comparability of m6A–ferroptosis studies.

## 5 Conclusion and outlook

The central insight from current evidence is that m6A modification regulates ferroptosis by controlling RNA fate at multiple levels. Writers, erasers, readers, RBPs, lncRNAs, circRNAs, exosomes and microenvironmental signals converge on a limited set of ferroptosis modules, especially cystine import, GPX4/FSP1 antioxidant defense, lipid activation, iron handling, autophagy and stress-response signaling. This convergence helps to reconcile the apparent complexity at the regulator level with the emerging coherent at the pathway level.

Although mechanistic evidence for the m6A–ferroptosis axis has expanded rapidly, its maturity remains uneven across targets and tumor contexts. Several regulator–target circuits have been supported across complementary experimental systems, whereas others remain defined mainly in restricted cellular or clinical settings. This heterogeneity partly explains why apparently similar m6A pathways do not always translate into consistent ferroptosis phenotypes or therapeutic responses across cancers.

Future research should prioritize several unresolved steps along the path from mechanism to translation. First, m6A–ferroptosis studies need to move beyond regulator-level associations toward transcript-specific mechanisms by defining the relevant methylated RNA, reader or eraser dependency, and resulting change in RNA fate. Second, ferroptosis causality should be established with functionally complementary readouts and rescue approaches, particularly when therapeutic conclusions are drawn from changes in individual ferroptosis-related proteins. Third, tumor heterogeneity should be addressed by determining which ferroptosis module is dominant in a given cellular, microenvironmental and treatment context; single-cell, spatial and multi-omics approaches may be particularly useful when coupled to functional validation. Fourth, translational development will require strategies that combine tumor-selective delivery with protection of ferroptosis-sensitive normal tissues and patient stratification based on active regulator–target circuits rather than regulator expression alone. Together, these priorities define a progression from identifying a methylated target, to establishing ferroptosis causality, to resolving tumor-specific dependency, and ultimately to testing whether the same molecular circuit can guide treatment selection in clinically relevant specimens.

## References

[B1] Bell H. N. Stockwell B. R. Zou W. (2024). Ironing out the role of ferroptosis in immunity. Immunity 57 (5), 941–956. 10.1016/j.immuni.2024.03.019 38749397 PMC11101142

[B2] Bersuker K. Hendricks J. M. Li Z. Magtanong L. Ford B. Tang P. H. (2019). The CoQ oxidoreductase FSP1 acts parallel to GPX4 to inhibit ferroptosis. Nature 575 (7784), 688–692. 10.1038/s41586-019-1705-2 31634900 PMC6883167

[B3] Bian Y. Xu S. Gao Z. Ding J. Li C. Cui Z. (2024). m(6)A modification of lncRNA ABHD11-AS1 promotes colorectal cancer progression and inhibits ferroptosis through TRIM21/IGF2BP2/FOXM1 positive feedback loop. Cancer Lett. 596, 217004. 10.1016/j.canlet.2024.217004 38838765

[B4] Chen D. Gu X. Nurzat Y. Xu L. Li X. Wu L. (2024). Writers, readers, and erasers RNA modifications and drug resistance in cancer. Mol. Cancer 23 (1), 178. 10.1186/s12943-024-02089-6 39215288 PMC11363509

[B5] Chen H. Xiao N. Zhang C. Li Y. Zhao X. Zhang R. (2025). JMJD6 K375 acetylation restrains lung cancer progression by enhancing METTL14/m6A/SLC3A2 axis mediated cell ferroptosis. J. Transl. Med. 23 (1), 233. 10.1186/s12967-025-06241-8 40011892 PMC11863413

[B6] Chen X. Du W. Wu J. Zhang L. Lu Y. Zhang X. (2026). Deletion of GPX8 enhances irradiation-induced ferroptosis through m(6)A hypomethylation-mediated upregulation of ACSL4 in oral cancer. Antioxid. Redox Signal 45 (4-6), 219–241. 10.1177/15230864261449406 42156324

[B7] Dai Z. Lin B. Qin M. Lin Y. Wang L. Liao K. (2025). METTL3-mediated m6A modification of SLC7A11 enhances nasopharyngeal carcinoma radioresistance by inhibiting ferroptosis. Int. J. Biol. Sci. 21 (4), 1837–1851. 10.7150/ijbs.100518 39990661 PMC11844296

[B8] Dai B. Li J. Xu L. Chen W. Chen J. Song M. (2026). YTHDF2-mediated stabilization of SREBF1 promotes lipid metabolic reprogramming and ferroptosis-associated radioresistance in Anaplastic thyroid carcinoma. Cancer Lett. 639, 218232. 10.1016/j.canlet.2025.218232 41435981

[B9] Deng X. Qing Y. Horne D. Huang H. Chen J. (2023). The roles and implications of RNA m(6)A modification in cancer. Nat. Rev. Clin. Oncol. 20 (8), 507–526. 10.1038/s41571-023-00774-x 37221357 PMC12466201

[B10] Deng L. Di Y. Chen C. Xia J. Lei B. Li N. (2024). Depletion of the N(6)-Methyladenosine (m6A) reader protein IGF2BP3 induces ferroptosis in glioma by modulating the expression of GPX4. Cell Death Dis. 15 (3), 181. 10.1038/s41419-024-06486-z 38429265 PMC10907351

[B11] Diao H. Tan H. Hu Y. Wang R. Cai P. Huang B. (2023). The m(6)A reader YTHDF1 promotes lung carcinoma progression via regulating ferritin mediate ferroptosis in an m(6)A-Dependent manner. Pharm. (Basel). 16 (2), 185. 10.3390/ph16020185 37259333 PMC9966794

[B12] Dixon S. J. Lemberg K. M. Lamprecht M. R. Skouta R. Zaitsev E. M. Gleason C. E. (2012). Ferroptosis: an iron-dependent form of nonapoptotic cell death. Cell 149 (5), 1060–1072. 10.1016/j.cell.2012.03.042 22632970 PMC3367386

[B13] Doll S. Proneth B. Tyurina Y. Y. Panzilius E. Kobayashi S. Ingold I. (2017). ACSL4 dictates ferroptosis sensitivity by shaping cellular lipid composition. Nat. Chem. Biol. 13 (1), 91–98. 10.1038/nchembio.2239 27842070 PMC5610546

[B14] Doll S. Freitas F. P. Shah R. Aldrovandi M. da Silva M. C. Ingold I. (2019). FSP1 is a glutathione-independent ferroptosis suppressor. Nature 575 (7784), 693–698. 10.1038/s41586-019-1707-0 31634899

[B15] Dominissini D. Moshitch-Moshkovitz S. Schwartz S. Salmon-Divon M. Ungar L. Osenberg S. (2012). Topology of the human and mouse m6A RNA methylomes revealed by m6A-seq. Nature 485 (7397), 201–206. 10.1038/nature11112 22575960

[B16] Ge W. Wang Q. Wang H. Zhang W. Chen L. Li Z. (2025). M6A-ALKBH5-dependent RBMS3-AS3 down-regulation suppresses ferroptosis to promote lung adenocarcinoma progression through HNRNPDL/ZEB1/GPX4 axis. NPJ Precis. Oncol. 9 (1), 356. 10.1038/s41698-025-01141-y 41249383 PMC12623742

[B17] Gong X. Wang J. Jiang A. Shao C. Jiang Y. Kuang Y. (2025). M(6)A modification mediates CACNA1A stability to drive the progression of ovarian cancer by inhibiting ferroptosis. J. Ovarian Res. 19 (1), 4. 10.1186/s13048-025-01907-9 41316396 PMC12771733

[B18] Han H. Li Y. Lin Z. Ma X. Huang W. Lu C. (2025). Exosomal miR-130a-3p confers cisplatin resistance in esophageal cancer by regulating ferroptosis via the suppression of METTL14-mediated m6A RNA methylation of FSP1. Int. Immunopharmacol. 146, 113804. 10.1016/j.intimp.2024.113804 39689599

[B19] He J. Dong C. Song X. Qiu Z. Zhang H. Jiang Y. (2025). Methyltransferase-like 7B participates in bladder cancer via ACSL3 m(6)A modification in a ferroptosis manner. Biol. Direct 20 (1), 9. 10.1186/s13062-025-00597-z 39833962 PMC11744867

[B20] Ji F. H. Fu X. H. Li G. Q. He Q. Qiu X. G. (2022). FTO prevents thyroid cancer progression by SLC7A11 m6A methylation in a ferroptosis-dependent manner. Front. Endocrinol. (Lausanne) 13, 857765. 10.3389/fendo.2022.857765 35721711 PMC9205202

[B21] Jia G. Fu Y. Zhao X. Dai Q. Zheng G. Yang Y. (2011). N6-methyladenosine in nuclear RNA is a major substrate of the obesity-associated FTO. Nat. Chem. Biol. 7 (12), 885–887. 10.1038/nchembio.687 22002720 PMC3218240

[B22] Jiang X. Stockwell B. R. Conrad M. (2021). Ferroptosis: mechanisms, biology and role in disease. Nat. Rev. Mol. Cell Biol. 22 (4), 266–282. 10.1038/s41580-020-00324-8 33495651 PMC8142022

[B23] Kagan V. E. Mao G. Qu F. Angeli J. P. Doll S. Croix C. S. (2017). Oxidized arachidonic and adrenic PEs navigate cells to ferroptosis. Nat. Chem. Biol. 13 (1), 81–90. 10.1038/nchembio.2238 27842066 PMC5506843

[B24] Ke J. Gao T. Shen Z. Li M. Zhang T. Wu D. (2025). Mechanism of LncRNA NORAD regulating ferroptosis in endometrial cancer cells by modifying GPX4 through FTO-Mediated m6A methylation. Cell Mol. Life Sci. 82 (1), 364. 10.1007/s00018-025-05855-x 41137882 PMC12553841

[B25] Kraft V. A. N. Bezjian C. T. Pfeiffer S. Ringelstetter L. Müller C. Zandkarimi F. (2020). GTP cyclohydrolase 1/Tetrahydrobiopterin counteract ferroptosis through lipid remodeling. ACS Cent. Sci. 6 (1), 41–53. 10.1021/acscentsci.9b01063 31989025 PMC6978838

[B26] Lei G. Zhuang L. Gan B. (2022). Targeting ferroptosis as a vulnerability in cancer. Nat. Rev. Cancer 22 (7), 381–396. 10.1038/s41568-022-00459-0 35338310 PMC10243716

[B27] Li L. Zeng J. He S. Yang Y. Wang C. (2024a). METTL14 decreases FTH1 mRNA stability via m6A methylation to promote sorafenib-induced ferroptosis of cervical cancer. Cancer Biol. Ther. 25 (1), 2349429. 10.1080/15384047.2024.2349429 38738555 PMC11093024

[B28] Li Y. Guo M. Qiu Y. Li M. Wu Y. Shen M. (2024b). Autophagy activation is required for N6-methyladenosine modification to regulate ferroptosis in hepatocellular carcinoma. Redox Biol. 69, 102971. 10.1016/j.redox.2023.102971 38056309 PMC10749285

[B29] Li Y. Jin H. Li Q. Shi L. Mao Y. Zhao L. (2024c). The role of RNA methylation in tumor immunity and its potential in immunotherapy. Mol. Cancer 23 (1), 130. 10.1186/s12943-024-02041-8 38902779 PMC11188252

[B30] Li M. Hu D. Tan C. Peng O. Yang Q. Feng Y. (2026a). An exosome-biomimetic photothermal nanocarrier for IGF2BP2 siRNA delivery and enhanced ferroptosis in head and neck squamous cell carcinoma. Mater. Today Bio 37, 102999. 10.1016/j.mtbio.2026.102999 PMC12996677 41859644

[B31] Li Y. Shen M. Li M. Qiu Y. Wang Y. Shao J. (2026b). m(6)A methylation dependent autophagy activation is required for anti-tumor effects of artesunate through ferroptosis in hepatocellular carcinoma. Chem. Biol. Interact. 427, 111950. 10.1016/j.cbi.2026.111950 41621698

[B32] Liu J. Yue Y. Han D. Wang X. Fu Y. Zhang L. (2014). A METTL3-METTL14 complex mediates Mammalian nuclear RNA N6-adenosine methylation. Nat. Chem. Biol. 10 (2), 93–95. 10.1038/nchembio.1432 24316715 PMC3911877

[B33] Liu L. Li H. Hu D. Wang Y. Shao W. Zhong J. (2022). Insights into N6-methyladenosine and programmed cell death in cancer. Mol. Cancer 21 (1), 32. 10.1186/s12943-022-01508-w 35090469 PMC8796496

[B34] Liu L. Ni Y. Yang Y. Zhang G. Mao S. Chao N. (2025a). Loss of NLN suppresses lung cancer progression by inducing ferroptosis through downregulating m(6)A methylation of GPX4. Redox Biol. 88, 103928. 10.1016/j.redox.2025.103928 41242017 PMC12664413

[B35] Liu X. Cui Y. Gong J. Yu X. Cui Y. Xuan Y. (2025b). SETD5 facilitates stemness and represses ferroptosis via m6A-mediating PKM2 stabilization in non-small cell lung cancer. Oncogene 44 (29), 2474–2489. 10.1038/s41388-025-03426-9 40307507

[B36] Ma L. Chen T. Zhang X. Miao Y. Tian X. Yu K. (2021a). The m(6)A reader YTHDC2 inhibits lung adenocarcinoma tumorigenesis by suppressing SLC7A11-dependent antioxidant function. Redox Biol. 38, 101801. 10.1016/j.redox.2020.101801 33232910 PMC7691619

[B37] Ma L. Zhang X. Yu K. Xu X. Chen T. Shi Y. (2021b). Targeting SLC3A2 subunit of system X(C)(-) is essential for m(6)A reader YTHDC2 to be an endogenous ferroptosis inducer in lung adenocarcinoma. Free Radic. Biol. Med. 168, 25–43. 10.1016/j.freeradbiomed.2021.03.023 33785413

[B38] Mao C. Liu X. Zhang Y. Lei G. Yan Y. Lee H. (2021). DHODH-Mediated ferroptosis defence is a targetable vulnerability in cancer. Nature 593 (7860), 586–590. 10.1038/s41586-021-03539-7 33981038 PMC8895686

[B39] Mao C. Jiang D. Koong A. C. Gan B. (2026). Exploiting metabolic cell death for cancer therapy. Nat. Rev. Cancer 26 (1), 27–45. 10.1038/s41568-025-00879-8 41094043

[B40] Meng X. Wang Z. Yang Q. Liu Y. Gao Y. Chen H. (2024). Intracellular C5aR1 inhibits ferroptosis in glioblastoma through METTL3-dependent m6A methylation of GPX4. Cell Death Dis. 15 (10), 729. 10.1038/s41419-024-06963-5 39368999 PMC11455874

[B41] Meyer K. D. Saletore Y. Zumbo P. Elemento O. Mason C. E. Jaffrey S. R. (2012). Comprehensive analysis of mRNA methylation reveals enrichment in 3' UTRs and near stop codons. Cell 149 (7), 1635–1646. 10.1016/j.cell.2012.05.003 22608085 PMC3383396

[B42] Mishima E. Nakamura T. Doll S. Proneth B. Fedorova M. Pratt D. A. (2025). Recommendations for robust and reproducible research on ferroptosis. Nat. Rev. Mol. Cell Biol. 26 (8), 615–630. 10.1038/s41580-025-00843-2 40204928

[B43] Qiao Y. Su M. Zhao H. Liu H. Wang C. Dai X. (2024). Targeting FTO induces colorectal cancer ferroptotic cell death by decreasing SLC7A11/GPX4 expression. J. Exp. Clin. Cancer Res. 43 (1), 108. 10.1186/s13046-024-03032-9 38600610 PMC11005233

[B44] Qu S. Feng B. Xing M. Qiu Y. Ma L. Yang Z. (2025). PRMT5 K240lac confers ferroptosis resistance via ALKBH5/SLC7A11 axis in colorectal cancer. Oncogene 44 (32), 2814–2830. 10.1038/s41388-025-03457-2 40447754

[B45] Ren H. Wang M. Ma X. An L. Guo Y. Ma H. (2024). METTL3 in cancer-associated fibroblasts-derived exosomes promotes the proliferation and metastasis and suppresses ferroptosis in colorectal cancer by eliciting ACSL3 m6A modification. Biol. Direct 19 (1), 68. 10.1186/s13062-024-00511-z 39160584 PMC11331890

[B46] Roshan M. K. Pal R. Kumari S. Kumar S. Muthuswamy S. (2025). The interplay of N6-Methyladenosine and ferroptosis in cancer: a promising therapeutic avenue. Cancer Genet. 296, 15–24. 10.1016/j.cancergen.2025.05.007 40482423

[B47] Roundtree I. A. Evans M. E. Pan T. He C. (2017). Dynamic RNA modifications in gene expression regulation. Cell 169 (7), 1187–1200. 10.1016/j.cell.2017.05.045 28622506 PMC5657247

[B48] Shi J. X. Zhang Z. C. Yin H. Z. Piao X. J. Liu C. H. Liu Q. J. (2024). RNA m6A modification in ferroptosis: implications for advancing tumor immunotherapy. Mol. Cancer 23 (1), 213. 10.1186/s12943-024-02132-6 39342168 PMC11437708

[B49] Song Z. Jia G. Ma P. Cang S. (2021). Exosomal miR-4443 promotes cisplatin resistance in non-small cell lung carcinoma by regulating FSP1 m6A modification-mediated ferroptosis. Life Sci. 276, 119399. 10.1016/j.lfs.2021.119399 33781830

[B50] Sun S. Gao T. Pang B. Su X. Guo C. Zhang R. (2022). RNA binding protein NKAP protects glioblastoma cells from ferroptosis by promoting SLC7A11 mRNA splicing in an m(6)A-dependent manner. Cell Death Dis. 13 (1), 73. 10.1038/s41419-022-04524-2 35064112 PMC8783023

[B51] Tian Q. Dan G. Wang X. Zhu J. Chen C. Tang D. (2025). IDO1 inhibits ferroptosis by regulating FTO-Mediated m6A methylation and SLC7A11 mRNA stability during glioblastoma progression. Cell Death Discov. 11 (1), 22. 10.1038/s41420-025-02293-3 39863603 PMC11762296

[B52] Wahida A. Conrad M. (2025). Decoding ferroptosis for cancer therapy. Nat. Rev. Cancer 25 (12), 910–924. 10.1038/s41568-025-00864-1 41073537

[B53] Wang X. Lu Z. Gomez A. Hon G. C. Yue Y. Han D. (2014). N6-methyladenosine-dependent regulation of messenger RNA stability. Nature 505 (7481), 117–120. 10.1038/nature12730 24284625 PMC3877715

[B54] Wang F. Sun Z. Zhang Q. Yang H. Yang G. Yang Q. (2023a). Curdione induces ferroptosis mediated by m6A methylation via METTL14 and YTHDF2 in colorectal cancer. Chin. Med. 18 (1), 122. 10.1186/s13020-023-00820-x 37735401 PMC10512537

[B55] Wang K. Wang G. Li G. Zhang W. Wang Y. Lin X. (2023b). m6A writer WTAP targets NRF2 to accelerate bladder cancer malignancy via m6A-dependent ferroptosis regulation. Apoptosis 28 (3-4), 627–638. 10.1007/s10495-023-01817-5 36719469

[B56] Wang Z. Li H. Cai H. Liang J. Jiang Y. Song F. (2023c). FTO sensitizes oral squamous cell carcinoma to ferroptosis via suppressing ACSL3 and GPX4. Int. J. Mol. Sci. 24 (22), 16339. 10.3390/ijms242216339 38003537 PMC10671523

[B57] Wang N. Chen H. Q. Zeng Y. Shi Y. Zhang Z. Li J. Y. (2024). Benzo(a)pyrene promotes the malignant progression of malignant-transformed BEAS-2B cells by regulating YTH N6-methyladenosine RNA binding protein 1 to inhibit ferroptosis. Toxicology 507, 153886. 10.1016/j.tox.2024.153886 39002880

[B58] Wang J. Yang J. Yao K. Tan J. Gao J. Zhou L. (2025). m6A-modified circCD2AP suppressed ferroptosis in bladder cancer by upregulating FOXC1 to promote PARK7 transcriptional activity. Chin. Med. J. Engl. 10.1097/cm9.0000000000003744 PMC13544742 40960159

[B59] Wang H. Jin Y. Dong Z. Hao J. Gao Z. Chen C. (2026). Methyltransferase-like 3/14-mediated m(6)A silencing of GPx3 drives lipophagy dysfunction and ferroptosis resistance in colorectal cancer. Res. (Wash D C) 9, 1273. 10.34133/research.1273 PMC13158459 42125307

[B60] Wu H. Chen S. Li X. Li Y. Shi H. Qing Y. (2025). RNA modifications in cancer. MedComm (2020) 6 (1), e70042. 10.1002/mco2.70042 39802639 PMC11718328

[B61] Xi S. Ming D. J. Zhang J. H. Guo M. M. Wang S. Y. Cai Y. (2023). Downregulation of N6-methyladenosine-modified LINC00641 promotes EMT, but provides a ferroptotic vulnerability in lung cancer. Cell Death Dis. 14 (6), 359. 10.1038/s41419-023-05880-3 37311754 PMC10264399

[B62] Xu Y. Lv D. Yan C. Su H. Zhang X. Shi Y. (2022). METTL3 promotes lung adenocarcinoma tumor growth and inhibits ferroptosis by stabilizing SLC7A11 m(6)A modification. Cancer Cell Int. 22 (1), 11. 10.1186/s12935-021-02433-6 34996469 PMC8742440

[B63] Xu L. Kong Y. Li K. Li J. Xu F. Xu Y. (2025). Neutrophil extracellular traps promote growth of lung adenocarcinoma by mediating the stability of m6A-mediated SLC2A3 mRNA-induced ferroptosis resistance and CD8(+) T cell inhibition. Clin. Transl. Med. 15 (2), e70192. 10.1002/ctm2.70192 39865544 PMC11769710

[B64] Yang W. S. SriRamaratnam R. Welsch M. E. Shimada K. Skouta R. Viswanathan V. S. (2014). Regulation of ferroptotic cancer cell death by GPX4. Cell 156 (1-2), 317–331. 10.1016/j.cell.2013.12.010 24439385 PMC4076414

[B65] Yang H. Hu Y. Weng M. Liu X. Wan P. Hu Y. (2022). Hypoxia inducible lncRNA-CBSLR modulates ferroptosis through m6A-YTHDF2-dependent modulation of CBS in gastric cancer. J. Adv. Res. 37, 91–106. 10.1016/j.jare.2021.10.001 35499052 PMC9039740

[B66] Yang R. Wan J. Ma L. Zhou F. Yang Z. Li Z. (2023). TMEM44-AS1 promotes esophageal squamous cell carcinoma progression by regulating the IGF2BP2-GPX4 axis in modulating ferroptosis. Cell Death Discov. 9 (1), 431. 10.1038/s41420-023-01727-0 38040698 PMC10692126

[B67] Yang H. Dong Y. Li R. Zhu L. Yang L. (2025a). LncRNA AC040169.1 is regulated by m6A modification and suppresses ferroptosis via SLC7A11 to promote ovarian cancer progression. J. Ovarian Res. 19 (1), 32. 10.1186/s13048-025-01922-w 41457273 PMC12857035

[B68] Yang Y. Jianxu Z. Hao L. Nini Q. Ge L. Jingjie Y. (2025b). EF24 targets METTL3 to reprogram m6A methylation and induce ferroptosis: an epitranscriptomic mechanism with therapeutical potential for glioma. Cell Commun. Signal 24 (1), 32. 10.1186/s12964-025-02583-4 41388311 PMC12817625

[B69] Yuan S. Xi S. Weng H. Guo M. M. Zhang J. H. Yu Z. P. (2023). YTHDC1 as a tumor progression suppressor through modulating FSP1-dependent ferroptosis suppression in lung cancer. Cell Death Differ. 30 (12), 2477–2490. 10.1038/s41418-023-01234-w 37903990 PMC10733405

[B70] Yuan C. Peng X. Tang H. Luo T. Jin M. Duan S. (2026). circSCYL2 antagonizes hnRNPA2B1-mediated m6A-chromatin crosstalk to promote ferroptosis and inhibit metastasis of breast cancer to brain. Int. J. Biol. Macromol. 343 (Pt 2), 149699. 10.1016/j.ijbiomac.2025.149699 41407226

[B71] Zaccara S. Ries R. J. Jaffrey S. R. (2019). Reading, writing and erasing mRNA methylation. Nat. Rev. Mol. Cell Biol. 20 (10), 608–624. 10.1038/s41580-019-0168-5 31520073

[B72] Zhang G. Mi W. Wang C. Li J. Zhang Y. Liu N. (2023). Targeting AKT induced ferroptosis through FTO/YTHDF2-dependent GPX4 m6A methylation up-regulating and degradating in colorectal cancer. Cell Death Discov. 9 (1), 457. 10.1038/s41420-023-01746-x 38102129 PMC10724184

[B73] Zhang C. Yang T. Chen H. Ding X. Chen H. Liang Z. (2025). METTL3 inhibition promotes radiosensitivity in hepatocellular carcinoma through regulation of SLC7A11 expression. Cell Death Dis. 16 (1), 9. 10.1038/s41419-024-07317-x 39799112 PMC11724875

[B74] Zhang Y. Meng T. Chen L. Li J. Li K. (2026). METTL3-mediated m(6)A modification contributes to anlotinib resistance in osteosarcoma by regulating ferroptosis via the circFAM120B/miR-330-3p/PRKDC axis. Free Radic. Biol. Med. 249, 409–423. 10.1016/j.freeradbiomed.2026.03.046 41839289

[B75] Zhao S. Cao J. Liang R. Peng T. Wu S. Liu Z. (2025). METTL16 suppresses ferroptosis in cholangiocarcinoma by promoting ATF4 via m(6)A modification. Int. J. Biol. Sci. 21 (1), 189–203. 10.7150/ijbs.97886 39744432 PMC11667817

[B76] Zheng G. Dahl J. A. Niu Y. Fedorcsak P. Huang C. M. Li C. J. (2013). ALKBH5 is a Mammalian RNA demethylase that impacts RNA metabolism and mouse fertility. Mol. Cell 49 (1), 18–29. 10.1016/j.molcel.2012.10.015 23177736 PMC3646334

[B77] Zheng J. Zhang Q. Zhao Z. Qiu Y. Zhou Y. Wu Z. (2023). Epigenetically silenced lncRNA SNAI3-AS1 promotes ferroptosis in glioma via perturbing the m(6)A-dependent recognition of Nrf2 mRNA mediated by SND1. J. Exp. Clin. Cancer Res. 42 (1), 127. 10.1186/s13046-023-02684-3 37202791 PMC10197824

[B78] Zhou Q. Meng Y. Li D. Yao L. Le J. Liu Y. (2024a). Ferroptosis in cancer: from molecular mechanisms to therapeutic strategies. Signal Transduct. Target Ther. 9 (1), 55. 10.1038/s41392-024-01769-5 38453898 PMC10920854

[B79] Zhou S. Liu J. Wan A. Zhang Y. Qi X. (2024b). Epigenetic regulation of diverse cell death modalities in cancer: a focus on pyroptosis, ferroptosis, cuproptosis, and disulfidptosis. J. Hematol. Oncol. 17 (1), 22. 10.1186/s13045-024-01545-6 38654314 PMC11040947

[B80] Zhou Y. Liu Z. Ma W. (2026). GDNF-AS1 mediated LHX2/METTL3/NCOA4 axis inhibits glioma progression via induction of ferroptosis. Neurochem. Int. 192, 106085. 10.1016/j.neuint.2025.106085 41275921

[B81] Zhu G. Xie Y. Bian Z. Ma J. Zhen N. Chen T. (2024). N6-methyladenosine modification of LATS2 promotes hepatoblastoma progression by inhibiting ferroptosis through the YAP1/ATF4/PSAT1 axis. Int. J. Biol. Sci. 20 (11), 4146–4161. 10.7150/ijbs.92413 39247829 PMC11379071

[B82] Zhu J. F. Guo D. P. Lv H. N. Liang Z. Y. Song J. Zeng W. (2025a). Histone lactylation-mediated up-regulation of IGF2BP2 enhances ferroptosis resistance via Nrf2 in colorectal cancer. Clin. Transl. Med. 15 (12), e70551. 10.1002/ctm2.70551 41399129 PMC12706171

[B83] Zhu Z. Xia X. Lu Y. Li D. He X. Zhang B. (2025b). PARK7-driven IGF2BP3-K76 lactylation mediates ferroptosis and HAIC resistance in hepatocellular carcinoma. Redox Biol. 87, 103869. 10.1016/j.redox.2025.103869 41038069 PMC12514540

[B84] Zhu C. Wu K. Wu T. Ma J. Ding B. Jiang N. (2026). WTAP-mediated glutaminase splicing bias suppresses ferroptosis in hepatocellular carcinoma. Cancer Commun. (Lond). 46, 0005. 10.34133/cancomm.0005 41727907 PMC12917114

[B85] Zou Y. Zheng S. Xie X. Ye F. Hu X. Tian Z. (2022). N6-methyladenosine regulated FGFR4 attenuates ferroptotic cell death in recalcitrant HER2-positive breast cancer. Nat. Commun. 13 (1), 2672. 10.1038/s41467-022-30217-7 35562334 PMC9106694

